# Limb-selective regions in the lateral temporal lobe shrink from childhood to adulthood

**DOI:** 10.1007/s00429-026-03152-2

**Published:** 2026-07-11

**Authors:** Selina Cohnen, Larissa Kahler, Seong Dae Yun, Kerstin Konrad, Marisa Nordt

**Affiliations:** 1 AG Developmental Cognitive Neuroscience, Institute of Medical Psychology and Medical Sociology, RWTH Aachen University, Pauwelsstraße 19, 52074 Aachen, Germany; 2 Forschungszentrum Jülich GmbH (INM-4), 52428 Jülich, Germany; 3 Forschungszentrum Jülich GmbH (INM-10), 52428 Jülich, Germany; 4 Institute for Medical Psychology and Medical Sociology, RWTH Aachen University, Pauwelsstraße 19, 52074 Aachen, Germany

**Keywords:** fMRI, Childhood development, Temporal cortex, Visual streams, Limb-selective regions, Body-selective regions

## Abstract

Perceiving hand gestures and inferring others’ actions and emotions from movements of hands and limbs play an important role in every-day interactions, especially in young children. The perception of categories such as limbs, bodies, or faces is supported by category-selective regions in the temporal lobe. Some category-selective regions, such as those reacting selectively to body parts or limbs, exist both on the ventral and on the lateral side of the temporal lobe, and are part of the ventral and lateral stream, respectively. While it was recently shown that limb-selective regions in the ventral stream shrink from childhood to adulthood, the developmental trajectory of limb-selective regions in the lateral stream remains unknown. To close this gap in knowledge, we acquired functional magnetic resonance imaging (fMRI) data in 21 children aged 10–12 years and 20 adults while they watched images of 10 visual categories including limbs and whole bodies. We first replicate the decrease of limb-selectivity from childhood to adulthood in the ventral temporal lobe. Across several analyses, our results further demonstrate that limb-selective regions in the lateral temporal lobe shrink as well, particularly in the left hemisphere. Underlining the specificity of our finding, we show that lateral body-selective regions show no significant development from childhood to adulthood. These findings advance our understanding of the developmental trajectories of limb- and body-selective regions and of the ventral and lateral visual streams more broadly.

**Supplementary Information:**

The online version contains supplementary material available at https://doi.org/10.1007/s00429-026-03152-2.

## Introduction

Long before children can speak fluently, they rely on their hands to guide others’ attention to objects of interest and to interpret the actions of those around them by watching their hands. For instance, around 9–12 months, infants understand and follow pointing gestures to interpret others’ communicative intentions and goals, and soon begin pointing themselves to create joint attention (Carpenter et al. 1998; Liszkowski et al. 2004; Tomasello et al. 2007). Likewise, in adulthood, hands and the gestures hands produce convey a wealth of socially relevant information about other individuals. This includes the actions they perform, the emotional states they are in or their intentions and thoughts. These examples highlight the importance of the perception of body parts, such as hands and limbs, for everyday social interactions.

High-level visual regions in the temporal lobe are critical for the perception of body parts (Downing et al. 2001; Peelen and Downing 2005). In fact, the temporal lobe contains functional regions responding preferentially to visual categories including body-parts or limbs (Downing et al. 2001; Peelen and Downing 2005; Weiner and Grill-Spector 2011), faces (Andrews and Ewbank 2004; Kanwisher et al. 1997; Puce et al. 1998), places (Epstein and Kanwisher 1998) and words (Dehaene et al. 2002). These regions are referred to as *category-selective regions*, because they show a stronger response to a specific category compared to other types of visual input. Critically, some category-selective regions are not confined to a single visual stream but are found in both the ventral and the lateral temporal lobe, as part of the ventral and lateral visual stream, respectively. The ventral stream extends from primary visual cortex (V1) to ventral temporal cortex (VTC) and supports perception and recognition (Grill-Spector et al. 2000; Moutoussis and Zeki 2002; Tong et al. 1998), whereas the lateral stream extends from V1 via areas involved in motion processing to the superior temporal sulcus (STS) and is thought to be involved in dynamic and social processing (Allison et al. 2000; Gandolfo et al. 2024; Gomez et al. 2019; Hein and Knight 2008; Pitcher and Ungerleider 2021; Weiner and Grill-Spector 2013) and action processing (Wurm and Caramazza 2022). The body-(part) selective region in the ventral stream is the fusiform body area (FBA; Peelen and Downing 2005), located in the occipito-temporal sulcus (OTS). In the lateral stream, the body-(part) selective region has been termed the extrastriate body area (EBA; Downing et al. 2001) consisting of three body-selective patches surrounding the motion selective region hMT+ (Weiner and Grill-Spector 2011).

How do these regions develop? Research on the development of body-selective regions has yielded mixed findings: One cross-sectional study reported increases in body-selectivity from childhood to adulthood in both ventral and lateral body-selective regions (Ross et al. 2014). In contrast, another cross-sectional study found no significant development of the FBA (Peelen et al. 2009), although the size of nearby face-selective regions increased across the same age range (Aylward et al. 2005; Cantlon et al. 2011; Golarai et al. 2007, 2010; Nordt et al. 2021; Peelen et al. 2009; Scherf et al. 2007). Results of this latter study were replicated by a recent longitudinal study showing that ventral regions responding selectively to whole bodies and limbs follow different developmental trajectories throughout childhood and adolescence (Nordt et al. 2021). Specifically, body-selective regions showed no developmental change, whereas limb-selective regions decreased in size from 5 to 17 years. Interestingly, limb selectivity in VTC decreased with age, while selectivity for words increased in the same region. These prolonged developments throughout childhood and adolescence suggest that neural resources originally dedicated to limb processing are repurposed to word recognition, a developmental mechanism described as “cortical recycling” (Dehaene and Cohen 2007; Nordt et al. 2021).

These results raise the question how limb-selective regions in the lateral stream develop from childhood to adulthood. Prior research suggests two hypotheses: One is that lateral limb-selective regions decrease in size with age, similar to the previously observed decrease in the ventral stream (Nordt et al. 2021). While it is unknown what is driving this development, a possible explanation might be that changes in limb-selectivity are linked to changing viewing behaviour for limbs (Kubota et al. 2024): That is, the visual diet – the distribution of visual inputs an individual sees in daily life – regarding the frequency of hands may change throughout childhood. In fact, studies using head-mounted cameras to record what children view, showed that hands occur highly frequently in the visual experience of infants and toddlers (Frank et al. 2012; Jayaraman et al. 2017; Yoshida and Smith 2008). In addition, recent eye-tracking studies indicate that viewing behaviour towards hands develops from childhood to adulthood: Preschool children allocate more visual attention to limbs when viewing naturalistic scenes, whereas literate adults preferentially attend to text (Linka et al. 2023, 2025). Such developmental changes in viewing behaviour may influence limb-selective responses in the ventral and lateral temporal lobe, leading to similar developmental trajectories across the ventral and lateral streams.

An alternative hypothesis suggested by prior literature is that lateral limb-selective regions remain stable. This view is supported by findings from Golarai and colleagues (2007), who showed that ventral face-selective regions expand from childhood to adulthood, whereas no such effects were found for lateral regions. In addition, the ventral and lateral stream differ with regard to the processing of static and dynamic stimuli (Pitcher et al. 2019), and these differences may affect the development of the two streams. Taken together, these results indicate that category-selective regions in the ventral and lateral stream follow different developmental trajectories and suggest that lateral limb-selective regions may show no developmental changes.

The decrease of limb-selectivity in the ventral stream also raises the question whether tool-selective regions (Bracci et al. 2012; Chao et al. 1999) may show a similar development. Notably, much like limb-selective regions, tool-selective regions have been reported both in the ventral and lateral temporal lobe: The ventral tool-selective region is located in the medial part of the fusiform gyrus (Bracci et al. 2016), the lateral tool-selective region is located in the left lateral occipitotemporal cortex (Peelen et al. 2013; Pillet et al. 2024). Two aspects suggest that tool-selective regions may undergo a developmental trajectory similar to that of limb-selective regions: First, tools, much like hands, manipulate objects and may often be viewed at the same time. Second, lateral tool-selective regions partially overlap with nearby located hand-selective regions in the brain (Bracci et al. 2012; Pillet et al. 2024). In fact, while a prior developmental study reported no significant development of tool-selectivity from childhood to adulthood (Dekker et al. 2011), they showed that children had additional ventral tool-selective clusters in the left fusiform gyrus compared to adults.

To address these questions, we collected fMRI data in 10-12-year-old children and adults and measured the size of their limb-selective regions in the ventral and lateral streams.

## Methods

### Statement on ethical regulations

The study was conducted in accordance with the Declaration of Helsinki and was approved by the Ethics Committee of the RWTH Aachen.

### Participants

Healthy participants with normal or corrected-to-normal vision and hearing were recruited from local institutions. Exclusion criteria were the diagnosis of a psychiatric or neurological disorder and/or a family history of dyslexia, and contraindications for MRI measurements, such as metallic implants, as assessed by a short non-standardized questionnaire.

21 children aged 10–12 years (11 female; mean age: *M* = 11.3 years; SD = 0.83) and 20 adults aged 22–61 years (17 female; mean age: *M* = 35.1 years; SD = 11.6) participated in this study. The age range for children was chosen because previous research demonstrated significant development in high-level visual regions from age 10 to adulthood (Golarai et al. 2010; Meissner et al. 2019; Nordt et al. 2019, 2021). The initial child sample consisted of 23 children (12 female; *M* = 11.31 years; SD = 0.84), but data of two children were excluded (one due to technical problems, one due to excessive head motion). All children reported German as their native language and two children spoke additional languages. 19 adults reported German as their native language, and one participant reported Russian as their native language but was fluent in German. Four children and two adults were left-handed as assessed by the Edinburgh Handedness Inventory (Oldfield 1971). Socioeconomic status, as well as race and ethnicity were not assessed. Most participants came from an urban or suburban environment in the regions Aachen and Cologne, Germany. The sample size in the present study is similar to those reported in previous studies on high-level visual cortex development (Dalski et al. 2026; Gomez et al. 2017). No statistical method was used to predetermine the sample size.

Testing took place at the Forschungszentrum Jülich (Germany). While adults participated in one session only, children took part in two sessions, which were conducted within a one-month period. This second session was introduced to compensate for possible data loss due to motion. If children already completed two runs with high scan quality in the first session (see below, Quality control of MRI Data), the runs of the first session were included in the analysis. Overall, each session lasted around 90–120 min. During the first session, children participated in a mock scanner training (see below, Mock-Scanner Training). Otherwise, the MRI scanning procedure was the same in both sessions. To prevent fatigue, behavioural testing was distributed over the two timepoints for the children. Adults participated in additional measurements as part of a larger study. Parents gave written consent and children gave verbal and/or written assent, adults gave written consent. Children were rewarded with a 10 Euro bookstore voucher and small toys of their choice for each session. Adults received 60 Euro.

### Behavioural testing

Outside the scanner participants performed a standardized reading test (Salzburger Lese- und Rechtschreibtest II, SLRT-II; Moll and Landerl 2010) in which they were instructed to read as many real words (task 1) and pseudowords (task 2) as possible in 60 seconds. All participants had scores in the normal range.

### Mock-scanner training

Children were prepared for their first MRI session with an extensive mock-scanner training to get familiarized with the scanner environment in a child-friendly way and to reduce motion during scanning (Raschle et al. 2012). During the training, children were introduced to scanner noises and practiced lying still. The importance of remaining still was underlined using examples of blurred versus sharp images (Raschle et al. 2012). The duration of the mock-scanner training was adapted to individuals’ needs and typically lasted between 20 and 30 minutes.

### MRI acquisition parameters

Structural and functional MRI data were collected at the Forschungszentrum Jülich on a 3-Tesla Siemens scanner using a 64-channel head coil.

A high-resolution T1-weighted structural MRI scan was acquired for anatomical imaging.

Structural imaging producing T1 contrast was acquired over a period of approximately 10 minutes with the following parameters: 2300ms TR, 2.32ms TE, 8° flip angle, 0.9 mm³ voxel size (isotropic). Additionally, a short T1-weighted anatomical “inplane scan” with identical slice orientation to the functional data was collected to facilitate alignment between the anatomical and the functional images with the software package mrVista (see below). Functional imaging (see below) comprised three separate runs, each lasting 5 minutes and 24 seconds. Imaging parameters of the functional sequences are 1000ms TR, 28.00ms TE, 60° flip angle, 2.5 mm³ voxel size leading to a slice thickness of 2.5 mm.

### fMRI paradigm: functional localizer

**Fig. 1:**
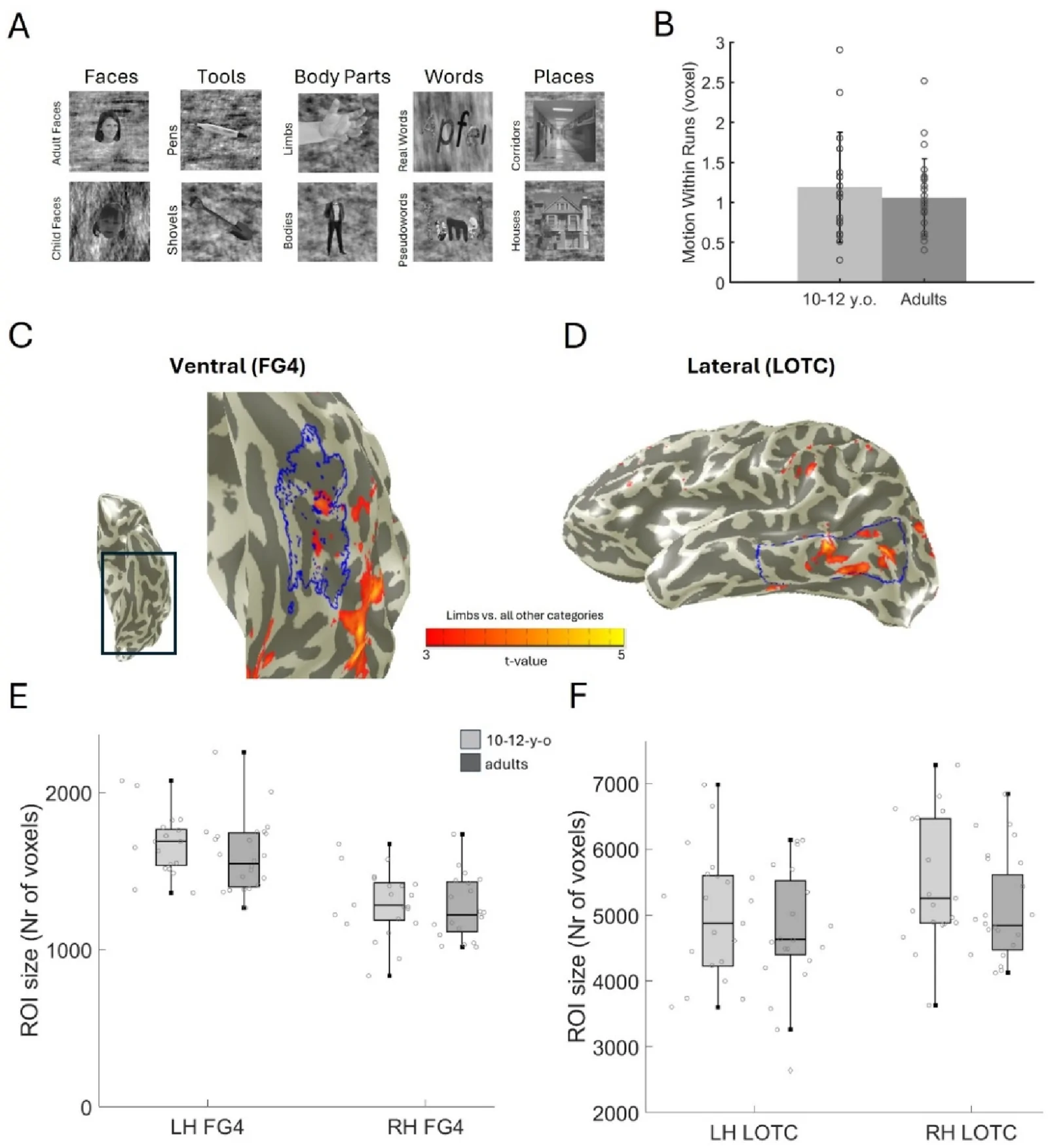
Experimental Stimuli, Quality Control and Example ROIs. **A** Stimuli presented to the participants during MRI scanning comprise five domains. (adapted from Stigliani et al. 2015): faces, tools, body parts, words, and places. Each domain includes two categories: child faces and adult faces, pens and shovels, headless bodies and limbs, real words and pseudowords, and houses and corridors. The faces illustrated were not presented in the real experiment and are the faces of two of the authors (M.N. and S.C), one of them as a child. **B** Mean within-run motion values (in voxels) during the included functional MRI runs for children (in light grey) and adults (in dark grey). **C** The ventral cytoarchitectonic ROI FG4 is outlined in blue on the ventral temporal cortex in the left hemisphere of a 10-year-old participant. The ventral limb-selective activation (defined by a t-value > 3 on the voxel-level for the contrast limbs vs. all other categories) falls within the FG4 ROI. **D** The anatomically defined LOTC ROI is outlined in blue on the lateral side of the left temporal lobe of an 11-year-old participant. The lateral limb-selective activation (defined by a t-value > 3 on the voxel-level for the contrast limbs vs. all other categories) falls within the LOTC ROI. **E** Sizes of the anatomical FG4 ROIs for children and adults in voxels. The horizontal line in each box denotes the median value. Whiskers extend to the most extreme data points that do not qualify as outliers. **F** Same as E, but for LOTC ROIs.

Participants performed three runs of a modified version of the fLoc-localizer (Stigliani et al. 2015). This modified version contains stimuli of five visual domains. Each domain includes two categories: faces (child faces, adult faces), bodies (headless bodies, limbs), places (corridors, houses), words (real words, pseudowords), and tools (shovels, pens; Fig. 1A). Specifically, we made the following changes to the stimuli of the original localizer: We used real German words instead of numbers and a different set of pseudowords, matched to the German real words. Further, we used tools instead of objects, and our limb stimuli did not contain any feet or legs, but only hands (some also showing arms). The remaining stimuli are the same as reported in prior publications (Gomez et al. 2019; Nordt et al. 2019, 2021, 2023; Stigliani et al. 2015). As in the original version of the localizer, stimuli were grey-scaled images and presented in 4-second blocks at a rate of 2 Hz. During scanning, participants were instructed to fixate on a small fixation cross and to perform an odd-ball task during which they were supposed to press a button whenever a scrambled image appeared.

### Quality control of MRI Data

Data were excluded if participants moved their head more than three functional voxels within a run or between two runs, as in prior publications (Dalski et al. 2024). As not all participants retained all three runs after applying this criterion, only two runs per participant were included in the analysis to ensure consistency across the samples. If all three runs were valid, the first two runs were chosen, as we expected participants to be more alert during the two initial runs. Applying these criteria, the average motion of included runs did not differ significantly between the groups (Fig. 1B).

### Processing of MRI data

For structural data, we used FreeSurfer’s (https://surfer.nmr.mgh.harvard.edu) “recon-all” pipeline to generate a cortical surface reconstruction of each participant.

For the functional data, analysis was performed in MATLAB R2019a (MathWorks, Inc.) using the mrVista software package (https://github.com/vistalab/vistasoft). Functional MRI data was aligned to each participant’s structural data in its’ native space. Motion correction was performed within and between functional runs. No spatial smoothing and no slice-timing correction were applied. Prior work using similar pipelines has adopted this strategy to preserve high spatial resolution and to avoid blurring of responses to different categories across anatomical boundaries (Gomez et al. 2017; Nordt et al. 2021, 2023). We transformed the time courses into percentage signal change by dividing each voxel’s data by the average response across the entire run. To estimate the contribution of each of the 10 conditions, a general linear model (GLM) was fit to each voxel by convolving the stimulus presentation design with the hemodynamic response function (HRF). We used the HRF as implemented in SPM (https://www.fil.ion.ucl.ac.uk/spm/).

### Anatomical regions of interest (ROIs)

Cytoarchitectonic regions of interest: To examine the development of limb-selectivity in the ventral stream, we leveraged the finding that the ventral limb-selective region consistently falls into the cytoarchitectonic region FG4 (Weiner et al. 2017). Cytoarchitectonic regions are characterized based on how their cells are organized. While they are identified in post-mortem tissue, a probabilistic estimate of the cytoarchitectonic areas of the ventral temporal lobe can be obtained from an atlas (Rosenke et al. 2018). This atlas includes the cytoarchitectonic regions FG1, FG2, FG3 and FG4 in VTC (Caspers et al. 2013; Lorenz et al. 2015). For this study, we focused on the FG4 ROI, since the ventral limb-selective region falls into this part of the brain (Fig. 1C). This relationship has been validated on children’s brains previously (Kubota et al. 2023).

Lateral occipitotemporal cortex (LOTC): To examine the development of limb-selectivity in the lateral stream, we defined lateral occipitotemporal cortex (LOTC) ROIs based on anatomical landmarks, as in prior studies (Bugatus et al. 2017; Weiner and Grill-Spector 2011) on the inflated cortical surface of each hemisphere in each participant (Fig. 1D). This ROI was chosen because prior research showed that the lateral limb-selective region falls into this anatomical ROI (Weiner and Grill-Spector 2013). The inferior border of the LOTC ROI was defined by the lateral border of the OTS, the superior border by the inferior side of the superior temporal sulcus (STS). The anterior border of the LOTC ROI was defined by the anterior tip of the mid fusiform sulcus (MFS), the posterior boundary was set posterior to the lateral occipital sulcus (LOS).

### Definition of category-selectivity and category-selective regions

To examine the development of category-selectivity, we applied two approaches: First, an observer-independent approach to examine the number of voxels selective to a given category within anatomically defined ROIs (FG4 and LOTC ROIs, see above, Cytoarchitectonic regions of interest; Lateral occipitotemporal cortex) in each participant. Second, we manually defined functional ROIs in each participant and examined their sizes. We used the same definition for category-selectivity at the voxel level across approaches: We contrasted the responses for a given category to those for all other categories (for instance, limbs vs. all other categories). A voxel was classified as category-selective for a threshold of t > 3 for the given contrast. This threshold was chosen because previous studies showed that category-selective regions can be defined reliably in children and adults using this threshold (Gomez et al. 2017; Natu et al. 2016; Nordt et al. 2021). In addition, to validate that our results do not depend on this specific threshold, we also conducted a threshold-independent analysis (see below).

Definition of category-selectivity in the anatomical ROIs. We used a data-driven and observer-independent approach to examine how category-selectivity develops in high-level visual cortex as in prior publications (Nordt et al. 2021). We assessed the selectivity to each category in anatomically defined ROIs (ventral FG4 and lateral LOTC ROIs) in each participant and counted the number of voxels that passed this threshold. We report both the relative number of category-selective voxels relative to the overall number of voxels within anatomical ROIs as well as absolute numbers of selective voxels (as supplemental analyses). Additionally, we conducted a threshold-independent analysis. For this, we calculated the t-value for a given contrast for each voxel in an anatomical ROI and then examined the mean t-value across all voxels.

Functional regions of interest (fROIs): We further manually delineated lateral limb-selective regions in both hemispheres on each participant’s native cortical surface by using a combination of functional and structural information as in previous studies (Nordt et al. 2021; Weiner and Grill-Spector 2011). Again, we used a threshold of t > 3 (voxel-level) for the definition of functional ROIs and the contrast “limbs vs. all other categories”. The lateral limb-selective region typically includes three limb-selective patches forming a crescent-shaped activation pattern around the motion-selective area hMT+ (Weiner and Grill-Spector 2011). Specifically, it consists of one activation patch on the lateral occipital sulcus/middle occipital gyrus located posterior to hMT+, the second patch on the middle temporal gyrus located anterior to hMT + and the third patch on the inferotemporal gyrus located inferior to hMT+. This limb-selective region overlaps partially with the extrastriate body area (EBA; Downing et al. 2001), which is typically defined using whole body stimuli.

### Statistical analyses

Statistical analyses were performed using MATLAB R2019a (MathWorks, Inc.). To determine whether there were significant differences between the groups, we first performed Levene’s test to control for the assumption of homogeneity of variances. If homogeneity of variances was present, we performed independent-samples t-tests, if not, Welch’s t-test was performed. Levene tests and results for all categories are reported in the supplements. Normal distribution was assumed, since the sample sizes for each group were *N* = 20 or larger. Significant effects were controlled for multiple comparisons (across the 10 categories in each ROI) using the Benjamini-Hochberg-FDR correction as implemented in MATLAB R2019a (MathWorks, Inc.).

## Results

### No group differences in the amount of motion during scanning or in the sizes of anatomically defined ROIs

We first tested whether children and adults differed significantly regarding their head motion during scanning. Independent samples t-tests revealed no significant differences between the two groups (t(39) = 0.712, *p* = 0.48) (Fig. 1B). Thus, differences in functional activation are unlikely to stem from motion differences. Next, we performed independent-samples t-tests to determine whether children and adults differed significantly in the sizes of the anatomically defined ROIs. There was no significant difference across groups for the FG4 ROIs (lh: t(39) = 1.06, *p* = 0.29; rh: t(39) = 0.52, *p* = 0.61, Fig. 1E) and no difference for the LOTC ROIs (lh: t(39) = 0.73, *p* = 0.47; rh: t(39) = 0.83, *p* = 0.41, Fig. 1F).

### Replication of the decrease of limb-selectivity in the ventral stream

**Fig. 2:**
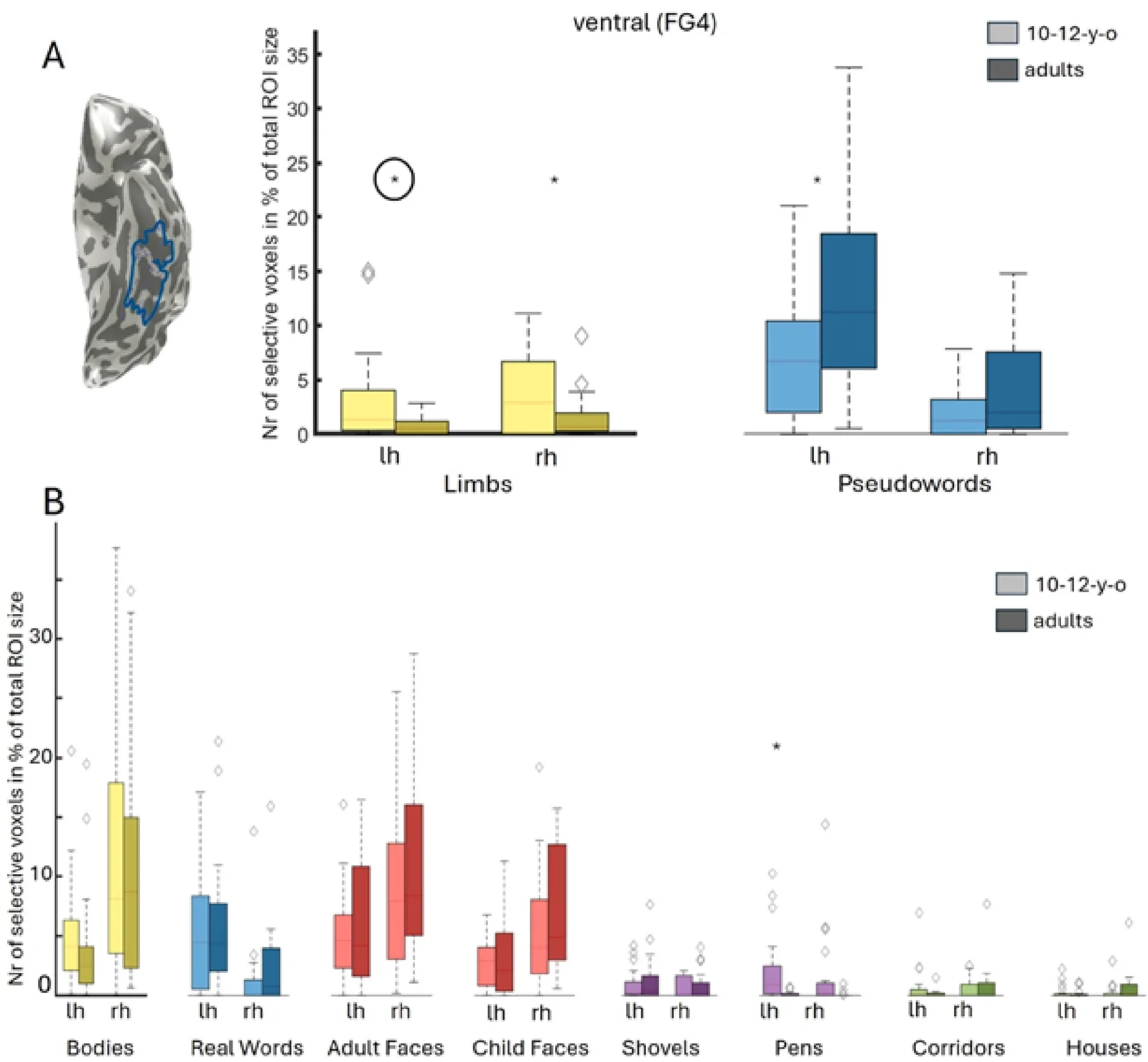
Development in ventral ROIs (FG4). **A** Left: Example showing the FG4 ROI in a 10-year-old participant. Right: Relative number of limb- and pseudoword-selective voxels in the ventral cytoarchitectonic FG4 ROI in 10-12-year-old children (brighter colours) and adults (darker colours). The horizontal line in each box denotes the median value. Whiskers extend to the most extreme data points that do not qualify as outliers. Diamonds are outliers, defined as lying beyond 1.5 interquartile ranges from the first or third quartile. Black asterisks indicate significant differences across the age groups, circles around asterisks indicate significant effects after correction for multiple comparisons for the 10 categories. **B** Same as A but for the categories bodies, real words, adult faces, child faces, shovels, pens, corridors and houses. Categories belonging to the same domain are displayed in the same colour

We first aimed to replicate previous findings reporting a decrease in limb-selectivity in the ventral temporal lobe (Nordt et al. 2021). Given that the ventral limb-selective region falls into the FG4 ROI (Kubota et al. 2023; Weiner et al. 2017), we tested whether the proportion of voxels within this ROI responding preferentially to limbs relative to other stimuli, differed between children and adults. Our results revealed that children had more limb-selective voxels compared to adults in the FG4 ROI in the left (t(39) = 2.63, *p* = 0.01, FDR-corrected *p* = 0.05, Welch’s t-test, Fig. 2A) and the right hemisphere (t(39) = 2.09, *p* = 0.04), although the latter effect did not survive correction for multiple comparisons for the 10 categories (FDR-corrected *p* = 0.21). In contrast, for whole bodies, no statistically significant differences between the groups in either the left (t(39) = 0.75, *p* = 0.46, Welch’s t-tests) or the right (t(39) = 0.67, *p* = 0.51) hemisphere were found, in line with prior results (Nordt et al. 2021; Peelen et al. 2009).

Since previous research revealed that decreases in limb-selectivity in VTC are linked to increases in pseudoword-selectivity (Nordt et al. 2021), we next examined the development of voxels selective to real- and pseudowords. Mirroring previous findings, we found a significant increase of the proportion of pseudoword-selective voxels in the FG4 ROI from childhood to adulthood in the left hemisphere (t(39)=-2.54, *p* = 0.02, FDR-corrected *p* = 0.07) which was trending after FDR-correction, but not in the right hemisphere (t(39)=-1.83, *p* = 0.08, Fig. 2A). Surprisingly, for real words, we found no significant development in neither the left (t(39)=-0.185, *p* = 0.86) nor the right (t(39)=-0.938 *p* = 0.35) hemisphere (Fig. 2B).

Interestingly, selectivity to pens, one of the tool categories, showed a similar trajectory to limb-selectivity. In fact, our results revealed a significant decrease of the number of voxels selective to pens from childhood to adulthood in the left (t(39) = 2.87, Welch’s t-test *p* = 0.01, FDR-corrected *p* = 0.05) but not the right FG4 ROI (t(39) = 1.98, Welch’s t-test *p* = 0.06, FDR-corrected *p* = 0.22; Fig. 2B). No such development was observed for shovels, the other tool category (lh: t (39)=-0.67, *p* = 0.51; rh: t(39)=-0.33, *p* = 0.74). Because ventral tool-selective regions lie in the medial fusiform gyrus (Bracci et al. 2016), these regions should be mainly located in the FG3 ROI. We therefore performed the same analysis in FG3 and found a significant decrease for pens (t(39) = 2.6, *p* = 0.017, Welch’s t-test) and for shovels (t(39) = 2.2, *p* = 0.035, Welch’s t-test) in the left but not in the right hemisphere (pens: t(39) = 1.58, *p* = 0.13, Welch’s t-test; shovels: t(39) = 1.63, *p* = 0.12, Welch’s t-test; Fig. S1). Thus, it is possible that the decrease in the number of pen-selective voxels in the left FG4 ROI is driven by tool-selective regions in FG3 that partially overlap with the FG4 ROI. Notably, the overall number of tool-selective voxels in both ROIs was very small.

We found no significant effect for any of the other categories (Fig. 2B; Tables S1, S2, S3**)**, including faces. This is in line with previous research because the FG4 ROI captures mFus-faces (FFA-2), but not pFus-faces (FFA-1), and only pFus-faces is expected to develop within the tested age range (Nordt et al. 2021).

### Selectivity to limbs decreases in the lateral stream

Next, we examined whether and how limb-selective regions develop in the lateral stream by using two methodological approaches: First, we manually delineated lateral limb-selective functional ROIs in each hemisphere of each participant. Second, we applied the same observer-independent approach as in the ventral stream: Here, we counted the proportion of limb-selective voxels relative to the overall number of voxels in anatomically defined LOTC ROIs. While the first approach captures the development of clustered limb-selective activation, the second approach (i) may also include non-clustered limb-selective activation, (ii) is observer-independent and (iii) serves as an internal replication.

We first examined the development of manually delineated lateral limb-selective regions which could be defined in 19 children in the left and in 20 in the right hemisphere, and in 20 adults in the left and 18 in the right hemisphere.

**Fig. 3:**
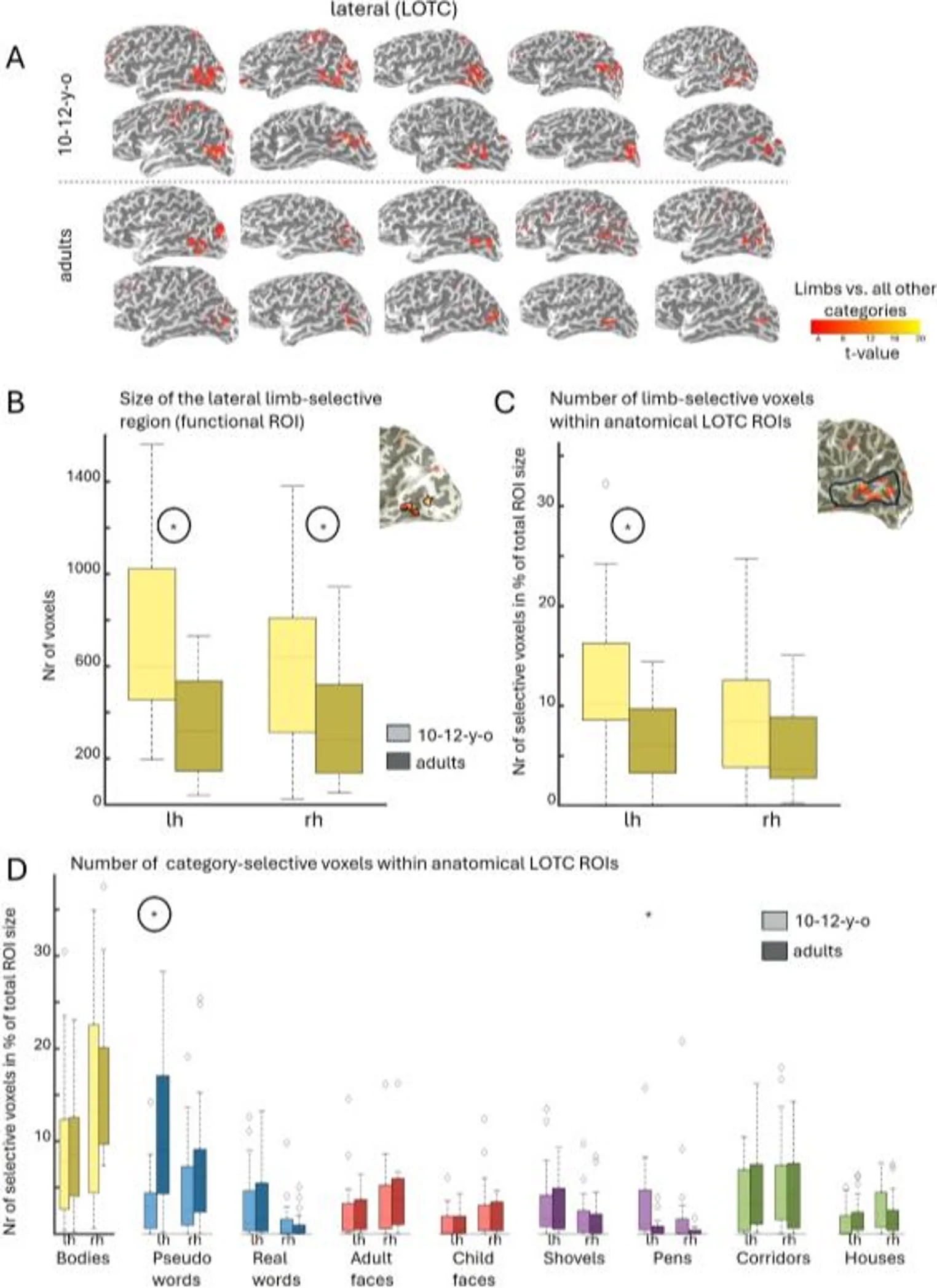
Development in lateral ROIs (LOTC). **A** The 10 children (upper rows) and adults (lower rows) with the highest number of limb-selective voxels in LOTC. Red patches depict limb-selective activation (limbs vs. all other stimuli, t > 3). Images of all participants can be found in the supplements. **B** Boxplots: Size of functional limb-selective ROIs (children: *N* = 19 in lh; *N* = 20 in rh; adults: *N* = 20 in lh; *N* = 18 in rh) in the lateral temporal lobe, measured in the number of voxels; Inset: Example for a lateral limb-selective region in the left hemisphere of an 11-year-old participant. **C** Boxplots: Relative number of limb-selective voxels out of all voxels in the left and the right anatomically defined LOTC ROI (children: *N* = 21 in lh and rh; adults: *N* = 20 in lh and rh); Inset: Example showing the limb-selective activation falling into the left LOTC ROI of an 11-year-old participant. **D** Same as boxplots in C but for bodies, pseudowords, real words, adult faces, child faces, shovels, pens, corridors and houses. Across all panels: Values of children are displayed in brighter colours and adults in darker colours. The horizontal line in each box denotes the median value. Whiskers extend to the most extreme data points that do not qualify as outliers. Diamonds are outliers, defined as lying beyond 1.5 interquartile ranges from the first or third quartile. Black asterisks indicate significant differences across the age groups, circles around asterisks indicate significant effects after correction for multiple comparisons for the 10 categories

Figure 3A displays the lateral view of the left hemispheres of the ten children (upper row) and ten adults (lower row) with the highest number of limb-selective voxels in the left lateral temporal lobe. Qualitative inspection of these maps suggests that children exhibit larger lateral limb-selective regions than adults (Fig. 3A; images of all participants can be found in Fig. S2). We next turned to quantitative analyses: This revealed larger limb-selective regions in children compared to adults both in the left (t(37) = 3.753, *p* = 0.0004, significant at the Bonferroni-corrected threshold (*p* = 0.0008) and right hemispheres (t(38) = 2.54, *p* = 0.01, Bonferroni-corrected *p* = 0.0245; Fig. 3B). The difference in the region’s size between groups was large: In children the left lateral limb-selective region had a mean size of 718 voxels (Median = 598, SD = 350), in adults this size was reduced to half the size with 357 voxels (Median = 329, SD = 212).

In the second methodological approach, we counted the proportion of limb-selective voxels within the LOTC ROIs. Results revealed a significant difference in the number of limb-selective voxels in the left hemisphere (t(39) = 2.74, *p* = 0.01, FDR-corrected *p* = 0.04; Fig. 3C), with children exhibiting a larger number of limb-selective voxels compared to adults. In the right hemisphere, this effect was only trending and did not survive correction for multiple comparisons (t(39) = 2.00, *p* = 0.05, FDR-corrected *p* = 0.39; Fig. 3C; all values in Tables S4, S5, S6). We repeated these analyses using the absolute number of limb-selective voxels within LOTC ROIs for internal replication, revealing a significant decrease in the left (t(39) = 2.88, *p* = 0.01, FDR-corrected *p* = 0.056) and right hemispheres (t(39) = 2.29, *p* = 0.03, FDR-corrected *p* = 0.139; Tables S7, S8, S9) that do not survive FDR correction.

To confirm that our results do not depend on a certain t-value used to determine category-selectivity, we additionally performed a threshold-independent analysis and compared the mean limb-selectivity across all voxels in the LOTC ROIs between the two groups. The selectivity value is calculated for each voxel within anatomically defined LOTC ROIs and is then averaged across all voxels. This analysis yielded a significant decrease of mean limb-selectivity from childhood to adulthood in the left (t(39) = 2.97 *p* = 0.01, FDR-corrected *p* = 0.05), but not the right LOTC ROI (t(39) = 1.42, *p* = 0.16; Fig. S3; Tables S10, S11, S12). In sum, these analyses show that limb-selective regions in the lateral stream shrink from childhood to adulthood and that this effect is more pronounced in the left compared to the right hemisphere.

As prior results demonstrated that the decrease of limb-selectivity in the ventral stream is directly linked to increases in selectivity to pseudowords (Nordt et al. 2021), we next tested whether the decrease in the number of limb-selective voxels in the lateral stream is paralleled by an increase in the number of voxels selective to another category. In particular, we reasoned that word-selective regions in the lateral temporal lobe, located on the inferior occipital sulcus (Stigliani et al. 2015; Zhan et al. 2023), may expand with development and partially overlap with our LOTC ROIs. In fact, we found that adults had a significantly larger number of voxels selective to pseudowords compared to children in the left (t(39)=-3.96, *p* = 0.0003, FDR-corrected *p* = 0.006, Welch’s t-test, Fig. 3D) but not right LOTC (t(39)=-1.42, *p* = 0.16, Welch’s t-test, Fig. 3D). This effect was specific to pseudowords, as no significant differences between the groups were observed for real words (lh: t(39)=-0.45, *p* = 0.65; rh: t(39) = 0.55, *p* = 0.58, Fig. 3C).

Specificity of the decrease of limb-selectivity in the lateral stream:

Finally, we tested the specificity of the decrease of limb-selectivity in the lateral stream. We asked whether the decrease in selectivity extends to two closely related categories including (i) whole body stimuli and (ii) tools, which are manipulated by hands. To do so, we first compared the relative number of body-selective voxels between the groups. Our results show no significant differences across age groups in either the left (t(39)=-0.34, *p* = 0.19) or the right (t(39)=-1.05, *p* = 0.96) hemisphere (Fig. 3D), suggesting that body- and limb-selective regions in the lateral temporal lobe follow different developmental trajectories. We next tested the development of the number of tool-selective voxels in the lateral stream. While we found a significantly larger number of voxels selective to pens in children compared to adults in the left hemisphere, this effect did not survive correction for multiple comparisons (t(39) = 2.6, Welch’s t-test *p* = 0.01, FDR-corrected *p* = 0.09; Fig. 3D) and was not significant in the right hemisphere (t(39) = 1.82, Welch’s t-test *p* = 0.08, FDR-corrected *p* = 0.31). No significant development was observed for the number of selective voxels for shovels from childhood to adulthood (Fig. 3D). We observed no significant development for any of the other categories (Tables S5, S6).

## Discussion

Our study has three main findings: First, we replicated the previously reported decrease of limb-selectivity in the ventral stream from childhood to adulthood (Nordt et al. 2021).

Second, we showed that this decrease also occurs in the lateral stream, where it was more pronounced in the left compared to the right hemisphere. Critically, this result was observed across different methodological approaches: (i) a functional ROI approach, (ii) an observer-independent approach, where we counted the number of limb-selective voxels within anatomically defined ROIs, and (iii) a threshold-independent approach, where we examined the mean t-value for limbs across all voxels in these anatomically defined ROIs. While the decrease of limb-selectivity in the left hemisphere was significant across all approaches, the decrease in the right hemisphere was only significant in the functional ROI approach. Third, we showed that across the ventral and lateral streams, the development of selectivity to limbs differed from that to whole bodies, corroborating prior findings (Nordt et al. 2021; Peelen et al. 2009).

In the following paragraphs we discuss the relationship between decreasing limb-selectivity and the development of selectivity for other categories, its possible causes, potential behavioural ramifications, the specificity of this finding and implications of this result for the development of visual streams, as well as the timing of the observed developmental effects.

A central question raised by our results is whether the observed changes in limb-selectivity might be linked to other developmental changes in the brain. Longitudinal results have revealed that decreases in limb-selectivity in the ventral stream were directly linked to increases in word-selectivity (Nordt et al. 2021) in line with the idea of a recycling of category-selectivity (Dehaene et al. 2010; Dehaene and Cohen 2007; Nordt et al. 2021). While the cross-sectional design of the present study precludes directly linking decreases in limb-selectivity to increases in word-selectivity in VTC, our results are consistent with this pattern: decreases in limb-selectivity were paralleled by increases in selectivity to pseudowords in the left hemisphere. This is further corroborated by a recent longitudinal study tracking children from before school onset through the end of their first school year, which demonstrates that as word-selectivity increases in OTS-word subregions, limb-selectivity decreases (Dalski et al. 2026).

In this context, the decrease of limb-selectivity and the parallel increase of pseudoword-selectivity observed in the lateral temporal lobe, raise the question whether these developments are linked as well. Consistent with this idea is the lateralization of the observed effects. Our results demonstrate a stronger and more consistent decrease of limb-selectivity in the left compared to the right LOTC, matching the left-lateralized increase in selectivity to pseudowords. Although our lateral LOTC ROI was not defined to capture a specific word-selective region, word-selectivity in our participants was substantial, and in many cases the word-selective patch located on the inferior occipital sulcus (Stigliani et al. 2015; Zhan et al. 2023) extended into the LOTC ROI. Thus, future longitudinal studies could test if this lateral word-selective patch may be engaged in cortical recycling. Critically, a decrease in limb-selectivity in lateral temporal cortex does not cast doubt on the recycling observed in VTC. Indeed, Nordt et al. (2021) already showed that decreases in limb-selectivity occurred not only within the expanding word-selective region, where decreases in limb-selectivity and increases in word-selectivity were directly linked, but also in other parts of VTC (e.g., in the right hemisphere). Therefore, a decrease in limb-selectivity in lateral temporal cortex may further demonstrate that such decreases can occur in broader parts of cortex, beyond the context of cortical recycling.

While it is currently unknown what drives the decrease of limb-selectivity across the ventral and lateral streams, our findings are in line with previous behavioural studies using eye-tracking to investigate differences in gaze behaviour between children and adults (Linka et al. 2023, 2025). These studies demonstrated that children look more at hands when freely viewing natural complex scenes, compared to adults, who look more at text. Thus, a possible explanation linking our findings with these behavioural results is that changes in viewing behaviour towards limbs from childhood to adulthood may affect category-selective responses to these stimuli in both the ventral (Kubota et al. 2024) and lateral stream, leading to a decrease of limb-selectivity. These developmental changes in viewing behaviour may contribute to the bilateral decrease in limb-selectivity. However, changes in the visual diet alone do not readily explain the stronger decrease of limb-selectivity observed in the left compared to the right hemisphere. In case of the ventral stream, we think that this hemispheric asymmetry reflects developmental processes related to the emergence of left-lateralized word-selective cortex and cortical recycling from limb- to word-selectivity. In fact, recent findings offer an additional explanation for the observed recycling from limbs to words in the ventral stream that is consistent with the lateralization of the observed effects (Dalski et al. 2026). They suggest that since younger children rely more heavily on their hands for communication than older children who have more advanced language skills, the parallel decrease in limb-selectivity and increase in word-selectivity may reflect a developmental shift in the extent to which a mapping of vision to (left-lateralized) language is required for processing these categories. Critically, the visual diet and vision-to-language mapping accounts are not mutually exclusive and may both contribute to cortical recycling in VTC. Future longitudinal studies combining measures of visual diet, vision-to-language mapping, and a characterization of high-level visual regions will be needed to elucidate the left-lateralization of the decrease in limb-selectivity in the lateral stream.

A related, interesting finding is that selectivity to pseudowords increased from childhood to adulthood in the left LOTC, but that no such increase was observed for real words. In fact, while adults showed higher activity to pseudowords compared to real words, children showed similar activity across different types of word stimuli. In adults, higher activity for pseudowords compared to real words (Dehaene et al. 2002) and for low-frequency words compared to high-frequency words (Kronbichler et al. 2004; Woolnough et al. 2020) in word-selective regions has been reported repeatedly. It is thought that this pattern of results may reflect easier activation of representations of frequently compared to infrequently used words (Kronbichler et al. 2004) and that pseudowords evoke the strongest activity because they require the longest search times in the neural lexicon (and ultimately do not match any representations in there; Woolnough et al. 2020). Thus, these results raise the possibility that activity for pseudowords in children is lower compared to adults because their neural lexicon is smaller and may lead to shorter search times.

Our analyses also shed light on the specificity of the decrease of limb-selectivity: We examined how the development of limb-selectivity relates to (i) other body stimuli and (ii) tools, which are manipulated by hands. While a prior developmental study suggested a decrease in size of the lateral right EBA from childhood to adulthood (Peelen et al. 2009), our results reveal no evidence for a developmental change for whole-body stimuli, mirroring previous work on the ventral stream (Nordt et al. 2021; Peelen et al. 2009). Further, we found that selectivity to pens, one of the tool categories, decreased from childhood to adulthood both in the ventral and in the lateral ROI. While this result may reflect differences in viewing frequency of this category across groups, it should be interpreted with caution, as the number of tool-selective voxels was overall small. Future studies using a variety of tool stimuli and high-field MRI may further investigate the possibility that limb- and tool-selective regions may share some developmental processes, which is in line with the finding that tool- and hand-selective regions partially overlap (Bracci et al. 2012; Pillet et al. 2024).

Our results also raise the question whether the decrease in limb-selectivity comes with behavioural consequences. For example, previous studies showed that increased face-selectivity was directly linked to higher performance on a face recognition task (Golarai et al. 2007) and word-selectivity in VTC to reading skills (Kubota et al. 2019). Yet, testing what may be the behavioural consequences of the decrease of limb-selectivity is complex. One reason for this is that the information derived from perceiving body parts like limbs plays a crucial role in several social-cognitive processes (Downing and Peelen 2016), which include gathering information on someone’s emotional state (Blythe et al. 2023), emphasizing speech, or to identify (object-directed) actions (Wurm and Schubotz 2017) or goals (Woodward 1998). Thus, future research is required to explore whether and how changes in limb-selectivity may relate to these different kinds of behaviours.

The present results also enhance our understanding of the development of different visual streams more broadly. Our results indicate that limb-selective regions develop similarly across the ventral and the lateral stream. As such, they differ from previous research showing a differential development of category-selective regions across streams (Golarai et al. 2007). Specifically, this research has shown that ventral, but not lateral face-selective regions expand with age, and the size of the ventral face-selective region was linked to face recognition skills (Golarai et al. 2007). In combination, these results suggest that for some category representations, development is stream-specific, while this is not the case for others. Nonetheless, it is also possible that the developmental trajectories of limb- and body-selective regions in the lateral and ventral streams may differ in earlier developmental periods, in line with findings showing that the development of the ventral stream is delayed compared to that of the dorsal stream (Bourne et al. 2024).

The age range of the present sample of children (10–12 years) is relevant when interpreting the timing of the observed developmental effects. First, these findings are consistent with evidence for a prolonged maturation of high-level visual cortex that extends beyond the age of 10 (Golarai et al. 2010; Nordt et al. 2021, 2023), and with research showing that neural systems supporting social attention and interactive processing continue to refine across middle childhood and adolescence (Oberwelland et al. 2016; Redcay and Warnell 2018). Second, these results underscore the importance of studying younger developmental samples. For example, fMRI work in infants suggests that the ventral body-selective area (FBA) may emerge later than the lateral body-selective area (EBA; Kosakowski et al. 2022). Future longitudinal research spanning infancy and the preschool years, and using both static and dynamic stimuli (Pitcher et al. 2019), will be crucial for delineating the developmental trajectories of limb- and body-selective regions across visual streams in early development.

## Supplementary Information

Below is the link to the electronic supplementary material.

Supplementary Material 1

## Data Availability

Aggregated data and code to generate the main figures will be made publicly available upon publication at https://github.com/selinacohnen/Limb-selective-regions-in-the-LTL. Raw (f)MRI data cannot be made publicly available because sharing is restricted under Institutional Review Board (IRB) regulations.

## References

[CR1] Allison T, Puce A, McCarthy G (2000) Social perception from visual cues: Role of the STS region. Trends Cogn Sci 4(7):267–278. 10.1016/S1364-6613(00)01501-1 10859571

[CR2] Andrews TJ, Ewbank MP (2004) Distinct representations for facial identity and changeable aspects of faces in the human temporal lobe. NeuroImage 23(3):905–913. 10.1016/j.neuroimage.2004.07.060 15528090

[CR3] Aylward EH, Park JE, Field KM, Parsons AC, Richards TL, Cramer SC, Meltzoff AN (2005) Brain Activation during Face Perception: Evidence of a Developmental Change. J Cogn Neurosci 17(2):308–319. 10.1162/0898929053124884 15811242

[CR4] Blythe E, Garrido L, Longo MR (2023) Emotion is perceived accurately from isolated body parts, especially hands. Cognition 230:105260. 10.1016/j.cognition.2022.105260 36058103

[CR5] Bourne JA, Cichy RM, Kiorpes L, Morrone MC, Arcaro MJ, Nielsen KJ (2024) Development of Higher-Level Vision: A Network Perspective. J Neurosci 44(40):e1291242024. 10.1523/JNEUROSCI.1291-24.2024 39358020 PMC11450542

[CR7] Bracci S, Cavina-Pratesi C, Ietswaart M, Caramazza A, Peelen MV (2012) Closely overlapping responses to tools and hands in left lateral occipitotemporal cortex. J Neurophysiol 107(5):1443–1456. 10.1152/jn.00619.2011 22131379

[CR6] Bracci S, Cavina-Pratesi C, Connolly JD, Ietswaart M (2016) Representational content of occipitotemporal and parietal tool areas. Neuropsychologia 84:81–88. 10.1016/j.neuropsychologia.2015.09.001 26344476

[CR8] Bugatus L, Weiner KS, Grill-Spector K (2017) Task alters category representations in prefrontal but not high-level visual cortex. NeuroImage 155:437–449. 10.1016/j.neuroimage.2017.03.062 28389381 PMC5518738

[CR9] Cantlon JF, Pinel P, Dehaene S, Pelphrey KA (2011) Cortical Representations of Symbols, Objects, and Faces Are Pruned Back during Early Childhood. Cereb Cortex 21(1):191–199. 10.1093/cercor/bhq078 20457691 PMC3000569

[CR10] Carpenter M, Nagell K, Tomasello M (1998) Social cognition, joint attention, and communicative competence from 9 to 15 months of age. Monogr Soc Res Child Dev, 63(4). https://www.scopus.com/pages/publications/17044452296 9835078

[CR11] Caspers J, Zilles K, Eickhoff SB, Schleicher A, Mohlberg H, Amunts K (2013) Cytoarchitectonical analysis and probabilistic mapping of two extrastriate areas of the human posterior fusiform gyrus. Brain Struct Function 218(2):511–526. 10.1007/s00429-012-0411-8 PMC3580145 22488096

[CR12] Chao LL, Haxby JV, Martin A (1999) Attribute-based neural substrates in temporal cortex for perceiving and knowing about objects. Nat Neurosci 2(10):913–919. 10.1038/13217 10491613

[CR13] Dalski A, Kular H, Jorgensen JG, Grill-Spector K, Grotheer M (2024) Both mOTS-words and pOTS-words prefer emoji stimuli over text stimuli during a lexical judgment task. Cereb Cortex 34(8):bhae339. 10.1093/cercor/bhae339 39191663 PMC11349430

[CR14] Dalski A, Schulz A, Klaes M, Pirsch M, Meinhardt M, Ukaj A, Grotheer M (2026) Preliterate symbolic language processing sets the neural stage for learning to read. bioRxiv, 2026–2005

[CR15] Dehaene S, Cohen L (2007) Cultural Recycling of Cortical Maps. Neuron 56(2):384–398. 10.1016/j.neuron.2007.10.004 17964253

[CR16] Dehaene S, Le Clec’H G, Poline J-B, Le Bihan D, Cohen L (2002) The visual word form area: A prelexical representation of visual words in the fusiform gyrus. NeuroReport 13(3):321–325. 10.1097/00001756-200203040-00015 11930131

[CR17] Dehaene S, Pegado F, Braga LW, Ventura P, Filho GN, Jobert A, Dehaene-Lambertz G, Kolinsky R, Morais J, Cohen L (2010) How Learning to Read Changes the Cortical Networks for Vision and Language. Science 330(6009):1359–1364. 10.1126/science.1194140 21071632

[CR19] Dekker T, Mareschal D, Sereno MI, Johnson MH (2011) Dorsal and ventral stream activation and object recognition performance in school-age children. NeuroImage 57(3):659–670. 10.1016/j.neuroimage.2010.11.005 21056677

[CR21] Downing PE, Peelen MV (2016) Body selectivity in occipitotemporal cortex: Causal evidence. Neuropsychologia 83:138–148. 10.1016/j.neuropsychologia.2015.05.033 26044771

[CR20] Downing PE, Jiang Y, Shuman M, Kanwisher N (2001) A Cortical Area Selective for Visual Processing of the Human Body. Science 293(5539):2470–2473. 10.1126/science.1063414 11577239

[CR22] Epstein R, Kanwisher N (1998) A cortical representation of the local visual environment. Nature 392(6676):598–601. 10.1038/33402 9560155

[CR23] Frank MC, Vul E, Saxe R (2012) Measuring the Development of Social Attention Using Free-Viewing. Infancy 17(4):355–375. 10.1111/j.1532-7078.2011.00086.x 32693486

[CR24] Gandolfo M, Abassi E, Balgova E, Downing PE, Papeo L, Koldewyn K (2024) Converging evidence that left extrastriate body area supports visual sensitivity to social interactions. Curr Biol 34(2):343–351e5. 10.1016/j.cub.2023.12.009 38181794

[CR25] Golarai G, Ghahremani DG, Whitfield-Gabrieli S, Reiss A, Eberhardt JL, Gabrieli JDE, Grill-Spector K (2007) Differential development of high-level visual cortex correlates with category-specific recognition memory. Nat Neurosci 10(4):512–522. 10.1038/nn1865 17351637 PMC3660101

[CR26] Golarai G, Liberman A, Yoon J, Grill-Spector K (2010) Differential development of the ventral visual cortex extends through adolescence. Front Hum Neurosci 3:1057 10.3389/neuro.09.080.2009 PMC2831628 20204140

[CR27] Gomez J, Barnett MA, Natu V, Mezer A, Palomero-Gallagher N, Weiner KS, Amunts K, Zilles K, Grill-Spector K (2017) Microstructural proliferation in human cortex is coupled with the development of face processing. Science 355(6320):68–71. 10.1126/science.aag0311 28059764 PMC5373008

[CR28] Gomez J, Drain A, Jeska B, Natu VS, Barnett M, Grill-Spector K (2019) Development of population receptive fields in the lateral visual stream improves spatial coding amid stable structural-functional coupling. NeuroImage 188:59–69. 10.1016/j.neuroimage.2018.11.056 30508682 PMC6413531

[CR29] Grill-Spector K, Kushnir T, Hendler T, Malach R (2000) The dynamics of object-selective activation correlate with recognition performance in humans. Nat Neurosci 3(8):837–843. 10.1038/77754 10903579

[CR30] Hein G, Knight RT (2008) Superior Temporal Sulcus—It’s My Area: Or Is It? J Cogn Neurosci 20(12):2125–2136. 10.1162/jocn.2008.20148 18457502

[CR31] Jayaraman S, Fausey CM, Smith LB (2017) Why are faces denser in the visual experiences of younger than older infants? Dev Psychol 53(1):38–49. 10.1037/dev0000230 28026190 PMC5271576

[CR32] Kanwisher N, McDermott J, Chun MM (1997) The fusiform face area: a module in human extrastriate cortex specialized for face perception. J Neurosci 17(11):4302–4311 9151747 10.1523/JNEUROSCI.17-11-04302.1997 PMC6573547

[CR33] Kosakowski HL, Cohen MA, Takahashi A, Keil B, Kanwisher N, Saxe R (2022) Selective responses to faces, scenes, and bodies in the ventral visual pathway of infants. Curr Biol 32(2):265–274e5. 10.1016/j.cub.2021.10.064 34784506 PMC8792213

[CR34] Kronbichler M, Hutzler F, Wimmer H, Mair A, Staffen W, Ladurner G (2004) The visual word form area and the frequency with which words are encountered: Evidence from a parametric fMRI study. NeuroImage 21(3). 10.1016/j.neuroimage.2003.10.021 15006661

[CR37] Kubota E, Joo SJ, Huber E, Yeatman JD (2019) Word selectivity in high-level visual cortex and reading skill. Dev Cogn Neurosci 36:100593. 10.1016/j.dcn.2018.09.003 30318344 PMC6969272

[CR36] Kubota E, Grotheer M, Finzi D, Natu VS, Gomez J, Grill-Spector K (2023) White matter connections of high-level visual areas predict cytoarchitecture better than category-selectivity in childhood, but not adulthood. Cereb Cortex 33(6):2485–2506. 10.1093/cercor/bhac221 35671505 PMC10016065

[CR35] Kubota E, Grill-Spector K, Nordt M (2024) Rethinking cortical recycling in ventral temporal cortex. Trends Cogn Sci 28(1):8–17. 10.1016/j.tics.2023.09.006 37858388 PMC10841108

[CR39] Linka M, Sensoy Ö, Karimpur H, Schwarzer G, De Haas B (2023) Free viewing biases for complex scenes in preschoolers and adults. Sci Rep 13(1):11803. 10.1038/s41598-023-38854-8 37479760 PMC10362043

[CR38] Linka M, Karimpur H, de Haas B (2025) Protracted development of gaze behaviour. Nat Hum Behav, 1–11 10.1038/s41562-025-02191-9 PMC12454113 40473802

[CR40] Liszkowski U, Carpenter M, Henning A, Striano T, Tomasello M (2004) Twelve-month‐olds point to share attention and interest. Dev Sci 7(3):297–307. 10.1111/j.1467-7687.2004.00349.x 15595371

[CR41] Lorenz S, Weiner KS, Caspers J, Mohlberg H, Schleicher A, Bludau S, Eickhoff SB, Grill-Spector K, Zilles K, Amunts K (2015) Two New Cytoarchitectonic Areas on the Human Mid-Fusiform Gyrus. Cereb Cortex bhv225. 10.1093/cercor/bhv225 PMC6248695 26464475

[CR42] Meissner TW, Nordt M, Weigelt S (2019) Prolonged functional development of the parahippocampal place area and occipital place area. NeuroImage 191:104–115. 10.1016/j.neuroimage.2019.02.025 30763610

[CR43] Moll K, Landerl K (2010) Salzburger Lese- und Rechtschreibtest II (SLRT-II). Hans Huber

[CR44] Moutoussis K, Zeki S (2002) The relationship between cortical activation and perception investigated with invisible stimuli. Proc Natl Acad Sci 99(14):9527–9532. 10.1073/pnas.142305699 12089336 PMC123174

[CR45] Natu VS, Barnett MA, Hartley J, Gomez J, Stigliani A, Grill-Spector K (2016) Development of Neural Sensitivity to Face Identity Correlates with Perceptual Discriminability. J Neurosci 36(42):10893–10907. 10.1523/JNEUROSCI.1886-16.2016 27798143 PMC5083016

[CR46] Nordt M, Gomez J, Natu V, Jeska B, Barnett M, Grill-Spector K (2019) Learning to Read Increases the Informativeness of Distributed Ventral Temporal Responses. Cereb Cortex 29(7):3124–3139. 10.1093/cercor/bhy178 30169753 PMC6611467

[CR47] Nordt M, Gomez J, Natu VS, Rezai AA, Finzi D, Kular H, Grill-Spector K (2021) Cortical recycling in high-level visual cortex during childhood development. Nat Hum Behav 5(12):1686–1697. 10.1038/s41562-021-01141-5 34140657 PMC8678383

[CR48] Nordt M, Gomez J, Natu VS, Rezai AA, Finzi D, Kular H, Grill-Spector K (2023) Longitudinal development of category representations in ventral temporal cortex predicts word and face recognition. Nat Commun 14(1):8010. 10.1038/s41467-023-43146-w 38049393 PMC10696026

[CR49] Oberwelland E, Schilbach L, Barisic I, Krall SC, Vogeley K, Fink GR, Herpertz-Dahlmann B, Konrad K, Schulte-Rüther M (2016) Look into my eyes: Investigating joint attention using interactive eye-tracking and fMRI in a developmental sample. NeuroImage 130:248–260. 10.1016/j.neuroimage.2016.02.026 26892856

[CR50] Oldfield RC (1971) The assessment and analysis of handedness: The Edinburgh Inventory. Neuropsychologia 9(1):97–113. 10.1016/0028-3932(71)90067-4 5146491

[CR52] Peelen MV, Downing PE (2005) Selectivity for the Human Body in the Fusiform Gyrus. J Neurophysiol 93(1):603–608. 10.1152/jn.00513.2004 15295012

[CR53] Peelen MV, Glaser B, Vuilleumier P, Eliez S (2009) Differential development of selectivity for faces and bodies in the fusiform gyrus. Dev Sci 12(6). 10.1111/j.1467-7687.2009.00916.x 19840035

[CR51] Peelen MV, Bracci S, Lu X, He C, Caramazza A, Bi Y (2013) Tool Selectivity in Left Occipitotemporal Cortex Develops without Vision. J Cogn Neurosci 25(8):1225–1234. 10.1162/jocn_a_00411 23647514

[CR54] Pillet I, Cerrahoğlu B, Philips RV, Dumoulin S, De Beeck O, H (2024) A 7T fMRI investigation of hand and tool areas in the lateral and ventral occipitotemporal cortex. PLoS ONE 19(11):e0308565. 10.1371/journal.pone.0308565 39499698 PMC11537398

[CR56] Pitcher D, Ungerleider LG (2021) Evidence for a Third Visual Pathway Specialized for Social Perception. Trends Cogn Sci 25(2):100–110. 10.1016/j.tics.2020.11.006 33334693 PMC7811363

[CR55] Pitcher D, Ianni G, Ungerleider LG (2019) A functional dissociation of face-, body- and scene-selective brain areas based on their response to moving and static stimuli. Sci Rep 9(1):8242. 10.1038/s41598-019-44663-9 31160680 PMC6546694

[CR57] Puce A, Allison T, Bentin S, Gore JC, McCarthy G (1998) Temporal Cortex Activation in Humans Viewing Eye and Mouth Movements. J Neurosci 18(6):2188–2199. 10.1523/JNEUROSCI.18-06-02188.1998 9482803 PMC6792917

[CR58] Raschle N, Zuk J, Ortiz-Mantilla S, Sliva DD, Franceschi A, Grant PE, Benasich AA, Gaab N (2012) Pediatric neuroimaging in early childhood and infancy: Challenges and practical guidelines. Ann N Y Acad Sci 1252(1):43–50. 10.1111/j.1749-6632.2012.06457.x 22524338 PMC3499030

[CR59] Redcay E, Warnell KR (2018) A Social-Interactive Neuroscience Approach to Understanding the Developing Brain. In *Advances in Child Development and Behavior* (Bd. 54, S. 1–44). Elsevier. 10.1016/bs.acdb.2017.10.001 29455860

[CR60] Rosenke M, Weiner KS, Barnett MA, Zilles K, Amunts K, Goebel R, Grill-Spector K (2018) A cross-validated cytoarchitectonic atlas of the human ventral visual stream. NeuroImage 170:257–270. 10.1016/j.neuroimage.2017.02.040 28213120 PMC5559348

[CR61] Ross PD, De Gelder B, Crabbe F, Grosbras M-H (2014) Body-selective areas in the visual cortex are less active in children than in adults. *Frontiers in Human Neuroscience*, *8*. 10.3389/fnhum.2014.00941 PMC4240043 25484863

[CR62] Scherf KS, Behrmann M, Humphreys K, Luna B (2007) Visual category-selectivity for faces, places and objects emerges along different developmental trajectories. Dev Sci 10(4). 10.1111/j.1467-7687.2007.00595.x 17552930

[CR63] Stigliani A, Weiner KS, Grill-Spector K (2015) Temporal Processing Capacity in High-Level Visual Cortex Is Domain Specific. J Neurosci 35(36):12412–12424. 10.1523/JNEUROSCI.4822-14.2015 26354910 PMC4563034

[CR64] Tomasello M, Carpenter M, Liszkowski U (2007) A New Look at Infant Pointing. Child Dev 78(3):705–722. 10.1111/j.1467-8624.2007.01025.x 17516997

[CR65] Tong F, Nakayama K, Vaughan JT, Kanwisher N (1998) Binocular Rivalry and Visual Awareness in Human Extrastriate Cortex. Neuron 21(4):753–759. 10.1016/S0896-6273(00)80592-9 9808462

[CR67] Weiner KS, Grill-Spector K (2011) Not one extrastriate body area: Using anatomical landmarks, hMT+, and visual field maps to parcellate limb-selective activations in human lateral occipitotemporal cortex. NeuroImage 56(4):2183–2199. 10.1016/j.neuroimage.2011.03.041 21439386 PMC3138128

[CR68] Weiner KS, Grill-Spector K (2013) Neural representations of faces and limbs neighbor in human high-level visual cortex: Evidence for a new organization principle. Psychol Res 77(1):74–97. 10.1007/s00426-011-0392-x 22139022 PMC3535411

[CR66] Weiner KS, Barnett MA, Lorenz S, Caspers J, Stigliani A, Amunts K, Zilles K, Fischl B, Grill-Spector K (2017) The Cytoarchitecture of Domain-specific Regions in Human High-level Visual Cortex. Cereb Cortex 27(1):146–161. 10.1093/cercor/bhw361 27909003 PMC5939223

[CR69] Woodward A (1998) Infants selectively encode the goal object of an actor’s reach. Cognition 69(1):1–34. 10.1016/S0010-0277(98)00058-4 9871370

[CR70] Woolnough O, Donos C, Rollo PS, Forseth KJ, Lakretz Y, Crone NE, Fischer-Baum S, Dehaene S, Tandon N (2020) Spatiotemporal dynamics of orthographic and lexical processing in the ventral visual pathway. Nat Hum Behav 5(3):389–398. 10.1038/s41562-020-00982-w 33257877 PMC10365894

[CR71] Wurm MF, Caramazza A (2022) Two ‘what’ pathways for action and object recognition. Trends Cogn Sci 26(2):103–116. 10.1016/j.tics.2021.10.003 34702661

[CR72] Wurm MF, Schubotz RI (2017) What’s she doing in the kitchen? Context helps when actions are hard to recognize. Psychon Bull Rev 24(2):503–509. 10.3758/s13423-016-1108-4 27383619

[CR73] Yoshida H, Smith LB (2008) What’s in View for Toddlers? Using a Head Camera to Study Visual Experience. Infancy 13(3):229–248. 10.1080/15250000802004437 20585411 PMC2888512

[CR74] Zhan M, Pallier C, Agrawal A, Dehaene S, Cohen L (2023) Does the visual word form area split in bilingual readers? A millimeter-scale 7-T fMRI study. SCIENCE ADVANCES 10.1126/sciadv.adf6140 PMC10075963 37018408

