# Supplementary material for: Limb-selective regions in the lateral temporal lobe shrink from childhood to adulthood
Source: Brain Struct Funct. 2026 Jul 11;231(7):95. doi: 10.1007/s00429-026-03152-2 (PMC13356069; doi:10.1007/s00429-026-03152-2)
Supplement: Supplementary file 1 — Supplementary Material 1 [file 429_2026_3152_MOESM1_ESM.pdf]

Supplementary Figures

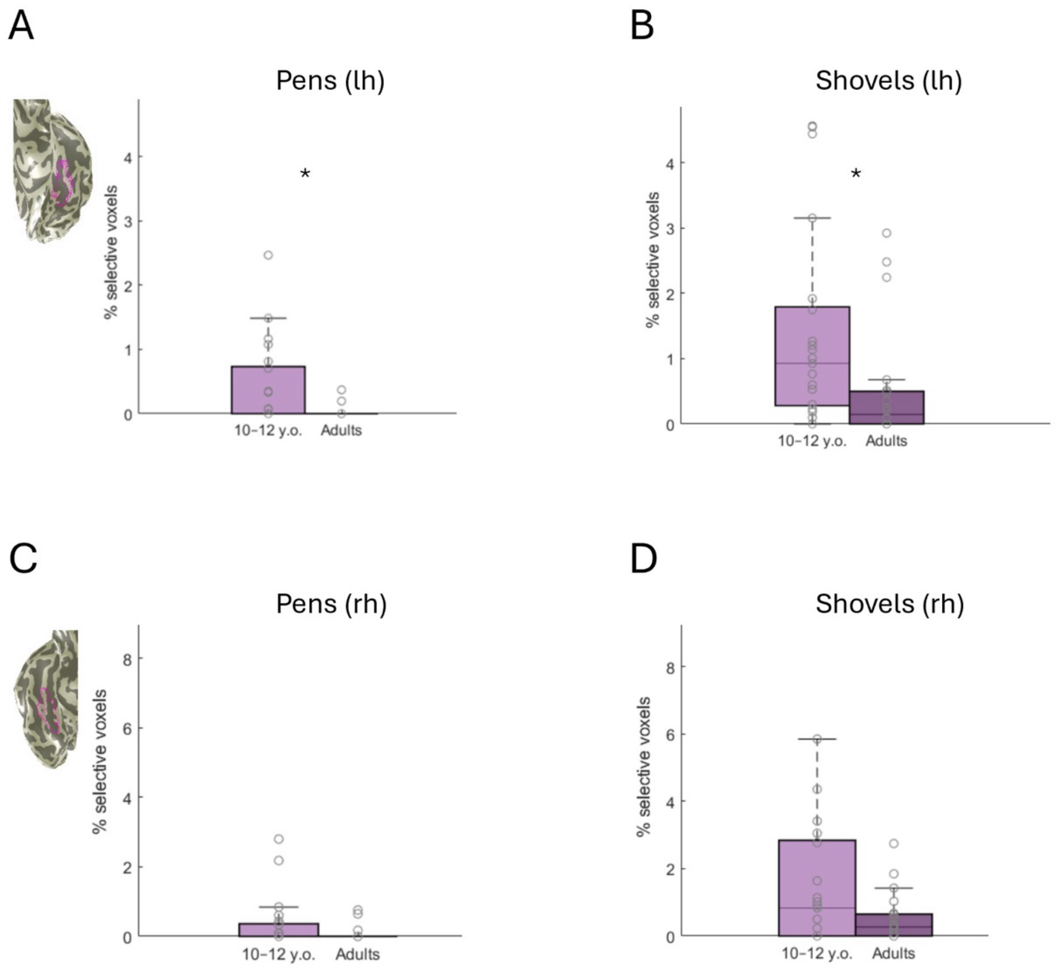

Supplementary Figure 1. A. Number of voxels in % out of all voxels in the FG3 ROI in the left hemisphere reacting selectively to pens. Inset: Example of a ventral FG3 ROI in the left hemisphere of an 11-year-old participant. The horizontal line in each box denotes the median value. Whiskers extend to the most extreme data points that do not qualify as outliers. Asterisks indicate a significant group difference ($p<0.05$). **B.** Same as A, but for shovels. **C.** Same as A, but for the right hemisphere. Inset: Example of a ventral FG3 ROI in the right hemisphere of an 11-year-old participant. **D.** Same as A, but for the right hemisphere and for shovels.

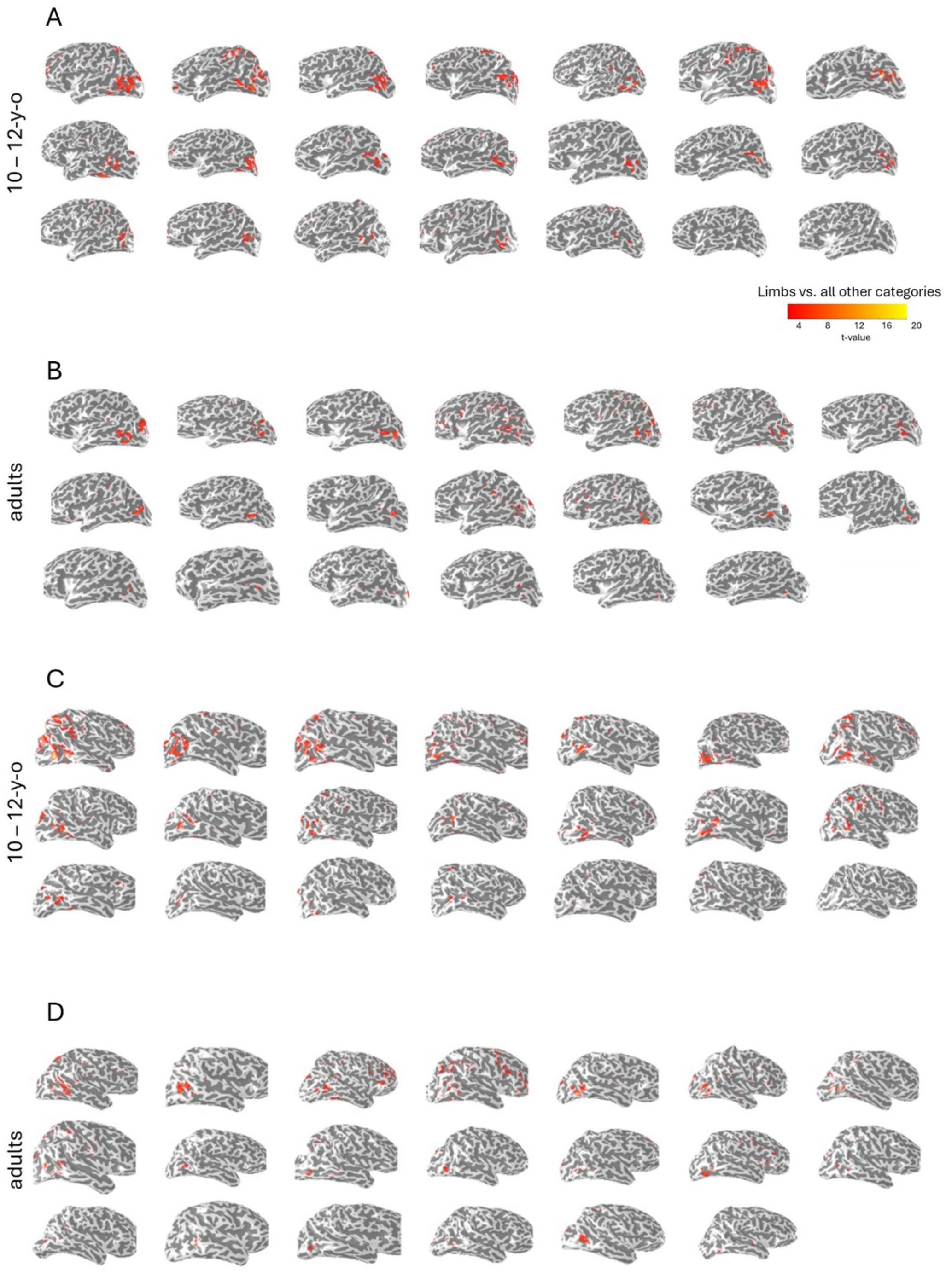

Supplementary Figure 2. A. Left inflated cortical surfaces of children sorted by the number of voxels reacting selectively to limbs relative to all voxels in the left LOTC ROI. Limb-selective patches are shown in red. **B.** Same as A, but for adults. **C.** Same as A, but for the right hemisphere. **D.** Same as A, but for the right hemisphere in adults.

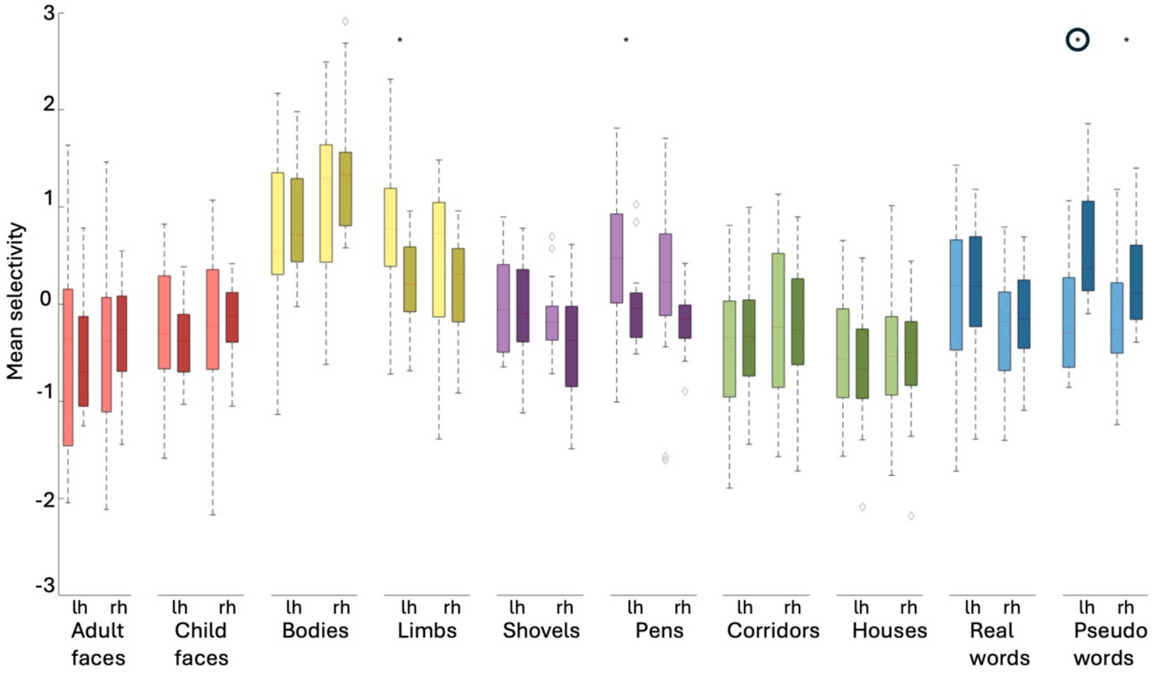

Supplementary Figure 3. Mean Selectivity in t-values across all voxels in the LOTC ROI. For each category, selectivity was computed by contrasting the responses to the given category to those of all other categories in the experiment. The horizontal line in each box denotes the median value. Whiskers extend to the most extreme data points that do not qualify as outliers. Diamonds are outliers, defined as lying beyond 1.5 interquartile ranges from the first or third quartile. Asterisks indicate a significant group difference ($p<0.05$). Circles around the asterisks indicate that the effect survives correction for multiple comparisons across the 10 categories.

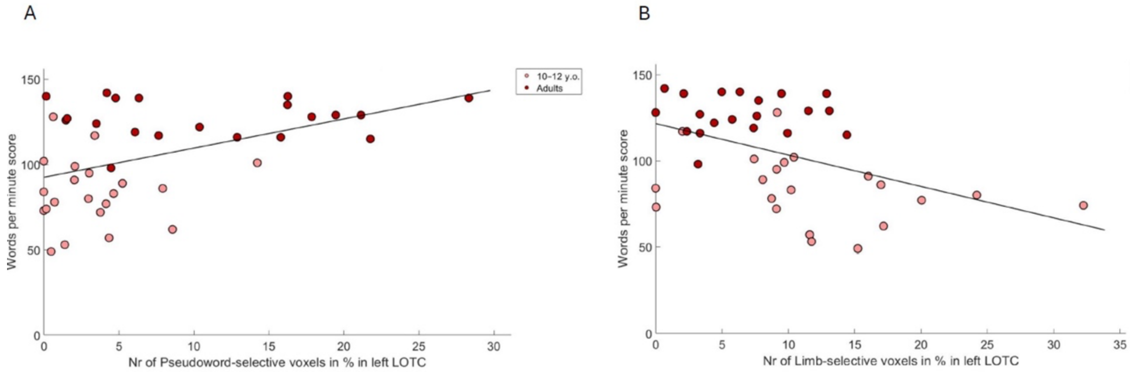

Supplementary Figure 4. A. Correlation between reading ability (the number of words read within one minute) and the relative number of voxels reacting selectively to pseudowords in the left LOTC ROI ($r = 0.454$; $p = 0.0029$). The correlation remains significant when age is accounted for (controlled for age $r = 0.349$; $p = 0.0275$). **B.** Correlation between reading ability (the number of words read within one minute) and the relative number of voxels reacting selectively to limbs in the left LOTC ROI ($r = -0.499$; $p = 0.0032$). When age is controlled for, the correlation is trending (controlled for age $r = 0.287$; $p = 0.0723$).

Supplementary Tables

Supplementary Table 1. Levene Test in the left (lh) and right (rh) hemispheres in the FG4 ROIs for the relative number of voxels selective to the respective category. Asterisks indicate that the Levene Test yielded significant results. If this was the case, Welch's T-Test was performed for the respective categories.

Category	Hemisphere	Levene Test (p-Value)
Adult Faces	lh	0.023*
Adult Faces	rh	0.116
Child Faces	lh	0.04
Child Faces	rh	0.558
Bodies	lh	0.043*
Bodies	rh	0.024*
Limbs	lh	0.002*
Limbs	rh	0.000*
Shovels	lh	0.171
Shovels	rh	0.209
Pens	lh	0.000*
Pens	rh	0.001*
Corridors	lh	0.014*
Corridors	rh	0.462
Houses	lh	0.09
Houses	rh	0.12
Words	lh	0.923
Words	rh	0.351
Pseudowords	lh	0.02*
Pseudowords	rh	0.004*

Supplementary Table 2. Independent samples t-tests or Welch's t-test determining whether the number of category-selective voxels (in % out of all voxels in the ROI) differ between children and adults in the FG4 ROI in the left hemisphere. Df=degrees of freedom. Bold p-values indicate the use of Welch's t-test. Asterisks indicate significant results of the t-tests.

Category	Hemisphere	df	T	p	FDR corrected p
Adult Faces	lh	39	-0.692	**0.493**	0.596
Child Faces	lh	39	-0.7	**0.488**	0.596
Bodies	lh	39	0.748	**0.459**	0.596
Limbs	lh	39	2.631	**0.012***	0.05
Shovels	lh	39	-0.673	0.505	0.561
Pens	lh	39	2.876	**0.007***	0.051
Corridors	lh	39	1.507	**0.14**	0.399
Houses	lh	39	0.851	0.4	0.561
Words	lh	39	-0.185	0.855	0.854
Pseudowords	lh	39	-2.542	**0.015***	0.07

Supplementary Table 3. Same as Supplementary Table 2 but for the right hemisphere.

Category	Hemisphere	df	T	p	FDR corrected p
Adult Faces	rh	39	-1.205	0.235	0.416
Child Faces	rh	39	-0.882	0.383	0.479
Bodies	rh	39	0.671	**0.506**	0.596
Limbs	rh	39	2.092	**0.043***	0.215
Shovels	rh	39	-0.33	0.744	0.743
Pens	rh	39	1.975	**0.055**	0.222
Corridors	rh	39	-0.477	0.636	0.707
Houses	rh	39	-1.168	0.25	0.416
Words	rh	39	-0.938	0.354	0.479
Pseudowords	rh	39	-1.831	**0.075**	0.249

Supplementary Table 4. Levene Test in the left (lh) and right (rh) hemispheres in the LOTC ROIs for the relative number of voxels selective to the respective category. Asterisks indicate that the Levene Test yielded significant results. If this was the case, Welch's T-Test was performed for the respective categories.

Category	Hemisphere	Levene Test (p-Value)
Adult Faces	lh	0.553
Adult Faces	rh	0.808
Child Faces	lh	0.549
Child Faces	rh	0.139
Bodies	lh	0.126
Bodies	rh	0.115
Limbs	lh	0.102
Limbs	rh	0.136
Shovels	lh	0.796
Shovels	rh	0.849
Pens	lh	0.000*
Pens	rh	0.009*
Corridors	lh	0.498
Corridors	rh	0.525
Houses	lh	0.353
Houses	rh	0.727
Words	lh	0.553
Words	rh	0.29
Pseudowords	lh	0.000*
Pseudowords	rh	0.308

Supplementary Table 5. Independent samples t-tests or Welch's t-tests determining whether the number of category-selective voxels (in % out of all voxels in the ROI) differ between children and adults in the LOTC ROI in the left hemisphere. Df=degrees of freedom. Bold p-values indicate the use of Welch's t-test. Asterisks indicate significant results of the t-tests.

Category	Hemisphere	df	T	p	FDR corrected p
Adult Faces	lh	39	0.191	0.85	0.875
Child Faces	lh	39	0.165	0.87	0.875
Bodies	lh	39	-0.341	0.19	0.475
Limbs	lh	39	2.739	0.009*	0.044
Shovels	lh	39	0.159	0.875	0.875
Pens	lh	39	2.599	**0.013**	0.088
Corridors	lh	39	-0.677	0.502	0.875
Houses	lh	39	-0.394	0.696	0.875
Words	lh	39	-0.454	0.652	0.875
Pseudowords	lh	39	-3.964	**0.0003**	0.006

Supplementary Table 6. Same as Supplementary Table 5, but for the right hemisphere.

Category	Hemisphere	df	T	p	FDR corrected p
Adult Faces	rh	39	-0.497	0.622	0.778
Child Faces	rh	39	0.997	0.325	0.759
Bodies	rh	39	-1.052	0.96	0.957
Limbs	rh	39	2.001	0.052	0.386
Shovels	rh	39	0.246	0.807	0.897
Pens	rh	39	1.815	**0.077**	0.309
Corridors	rh	39	0.710	0.482	0.778
Houses	rh	39	0.889	0.38	0.759
Words	rh	39	0.554	0.583	0.778
Pseudowords	rh	39	-1.418	0.164	0.547

Supplementary Table 7. Levene Test in the left (lh) and right (rh) hemispheres in the LOTC ROIs for the absolute number of voxels selective to the respective category. Asterisks indicate that the Levene Test yielded significant results. If this was the case, Welch's T-Test was performed for the respective categories.

Category	Hemisphere	Levene Test (p-Value)
Adult Faces	lh	0.337
Adult Faces	rh	0.493
Child Faces	lh	0.434
Child Faces	rh	0.1
Bodies	lh	0.041*
Bodies	rh	0.03*
Limbs	lh	0.04*
Limbs	rh	0.067
Shovels	lh	0.862
Shovels	rh	0.789
Pens	lh	0.000*
Pens	rh	0.004*
Corridors	lh	0.551
Corridors	rh	0.678
Houses	lh	0.268
Houses	rh	0.506
Words	lh	0.261
Words	rh	0.284
Pseudowords	lh	0.002*
Pseudowords	rh	0.2

Supplementary Table 8. Independent samples t-tests or Welch's t-tests determining whether the absolute number of voxels in the ROI differs between children and adults in the LOTC in the left hemisphere. Df=degrees of freedom. Bold p-values indicate the use of Welch's t-test. Asterisks indicate significant results of the t-tests.

Category	Hemisphere	df	T	p	FDR corrected p
Adult Faces	lh	39	0.359	0.722	0.932
Child Faces	lh	39	0.206	0.838	0.932
Bodies	lh	39	-0.086	**0.932**	0.932
Limbs	lh	39	2.879	**0.006***	0.056
Shovels	lh	39	0.104	0.918	0.932
Pens	lh	39	2.778	**0.008***	0.056
Corridors	lh	39	-0.539	0.593	0.932
Houses	lh	39	-0.477	0.636	0.932
Words	lh	39	-0.569	0.573	0.932

Pseudowords	lh	39	-3.663	**0.001***	0.015

Supplementary Table 9. Same as Supplementary Table 8, but for the right hemisphere.

**Category**	**Hemisphere**	**df**	**T**	**p**	**FDR corrected p**
Adult Faces	rh	39	-0.172	0.864	0.932
Child Faces	rh	39	1.055	0.298	0.745
Bodies	rh	39	-0.423	**0.675**	0.932
Limbs	rh	39	2.286	0.028*	0.139
Shovels	rh	39	0.281	0.78	0.932
Pens	rh	39	1.982	**0.055**	0.218
Corridors	rh	39	0.913	0.367	0.815
Houses	rh	39	1.129	0.266	0.745
Words	rh	39	0.585	0.562	0.932
Pseudowords	rh	39	-1.467	0.15	0.502

Supplementary Table 10. Levene Test in the left (lh) and right (rh) hemispheres in the LOTC ROIs for the mean selectivity (in t-values averaged across all voxels in the ROI). Asterisks indicate that the Levene Test yielded significant results. If this was the case, Welch's T-Test was performed for the respective categories.

**Category**	**Hemisphere**	**Levene Test (p-Value)**
Adult Faces	lh	0.04*
Adult Faces	rh	0.026*
Child Faces	lh	0.044*
Child Faces	rh	0.007*
Bodies	lh	0.125
Bodies	rh	0.066
Limbs	lh	0.224
Limbs	rh	0.099
Shovels	lh	0.618
Shovels	rh	0.026*
Pens	lh	0.028*
Pens	rh	0.01*
Corridors	lh	0.863
Corridors	rh	0.3
Houses	lh	0.703
Houses	rh	0.51
Words	lh	0.354
Words	rh	0.33

Pseudowords	lh	0.654
Pseudowords	rh	0.787

Supplementary Table 11. Independent samples t-tests or Welch's t-tests determining whether the mean selectivity (in t-values averaged across all voxels in the ROI) differs between children and adults in the LOTC ROI in the left hemisphere. Df=degrees of freedom. Bold p-values indicate the use of Welch's t-test. Asterisks indicate significant results of the t-tests.

**Category**	**Hemisphere**	**df**	**T**	**p**	**FDR corrected p**
Adult Faces	lh	39	0.369	**0.714**	0.841
Child Faces	lh	39	0.049	**0.961**	0.961
Bodies	lh	39	-0.743	0.462	0.841
Limbs	lh	39	2.974	0.005*	0.05
Shovels	lh	39	0.58	0.566	0.841
Pens	lh	39	2.21	**0.01***	0.167
Corridors	lh	39	-0.727	0.472	0.841
Houses	lh	39	0.641	0.525	0.841
Words	lh	39	-0.371	0.713	0.841
Pseudowords	lh	39	-4.55	0.0003*	0.001

Supplementary Table 12. Same as Supplementary Table 11, but for the right hemisphere.

**Category**	**Hemisphere**	**df**	**T**	**p**	**FDR corrected p**
Adult Faces	rh	39	-0.405	**0.688**	0.841
Child Faces	rh	39	-0.453	**0.653**	0.841
Bodies	rh	39	-1.046	0.302	0.755
Limbs	rh	39	1.418	0.164	0.511
Shovels	rh	39	1.37	**0.179**	0.511
Pens	rh	39	1.906	**0.08**	0.256
Corridors	rh	39	0.253	0.801	0.844
Houses	rh	39	0.31	0.758	0.842
Words	rh	39	-0.758	0.453	0.841
Pseudowords	rh	39	-2.372	0.023*	0.151
